# 3,3′-(2-Oxocyclo­pentane-1,3-di­yl)dipropane­nitrile

**DOI:** 10.1107/S1600536807068535

**Published:** 2008-01-09

**Authors:** Yi Deng, Yan-Xue Chen, Zhi-Lei Gao, Jin-Hui Yang, Wei Wang

**Affiliations:** a College of Pharmaceuticals & Biotechnology, Tianjin University, Tianjin 300072, People’s Republic of China; b School of Chemical Engineering and Technology, Tianjin University, Tianjin 300072, People’s Republic of China; c School of Materials Science and Engineering, Shijizhuang Railway Institute, Shijiazhuang 050043, People’s Republic of China

## Abstract

The complete mol­ecule of the title compound, C_11_H_14_N_2_O, is generated by crystallographic twofold symmetry, with the C=O group lying on the rotation axis. In the crystal structure, weak C—H⋯N inter­actions form zigzag chains of mol­ecules.

## Related literature

For the synthesis, see: Westman & Kober (1964 ▶). For a similar compound, see: Chen *et al.* (2007 ▶).
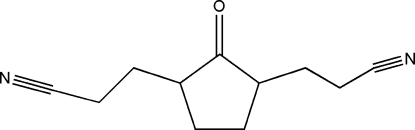


## Experimental

### 

#### Crystal data

C_11_H_14_N_2_O
*M* *_r_* = 190.24
Monoclinic, 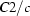

*a* = 18.261 (3) Å
*b* = 7.8182 (10) Å
*c* = 8.1943 (11) Å
β = 111.510 (9)°
*V* = 1088.4 (3) Å^3^
*Z* = 4
Mo *K*α radiation
μ = 0.08 mm^−1^
*T* = 294 (2) K
0.24 × 0.20 × 0.10 mm

#### Data collection

Bruker SMART CCD diffractometer
Absorption correction: multi-scan (*SADABS*; Bruker, 1997 ▶) *T*_min_ = 0.971, *T*_max_ = 0.992
3003 measured reflections
1114 independent reflections
644 reflections with *I* > 2σ(*I*)
*R*_int_ = 0.045

#### Refinement

*R*[*F* ^2^ > 2σ(*F* ^2^)] = 0.048
*wR*(*F* ^2^) = 0.133
*S* = 1.03
1114 reflections
65 parameters
H-atom parameters constrained
Δρ_max_ = 0.15 e Å^−3^
Δρ_min_ = −0.13 e Å^−3^

### 

Data collection: *SMART* (Bruker, 1997 ▶); cell refinement: *SAINT* (Bruker, 1997 ▶); data reduction: *SAINT*; program(s) used to solve structure: *SHELXS97* (Sheldrick, 2008 ▶); program(s) used to refine structure: *SHELXL97* (Sheldrick, 2008 ▶); molecular graphics: *SHELXTL* (Bruker, 1997 ▶); software used to prepare material for publication: *SHELXTL*).

## Supplementary Material

Crystal structure: contains datablocks I, global. DOI: 10.1107/S1600536807068535/hb2685sup1.cif

Structure factors: contains datablocks I. DOI: 10.1107/S1600536807068535/hb2685Isup2.hkl

Additional supplementary materials:  crystallographic information; 3D view; checkCIF report

## Figures and Tables

**Table 1:** Hydrogen-bond geometry (Å, °)

*D*—H⋯*A*	*D*—H	H⋯*A*	*D*⋯*A*	*D*—H⋯*A*
C5—H5*B*⋯N1^i^	0.97	2.54	3.466 (3)	160

Symmetry code: (i) 
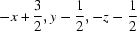
.

**Fig. 1.**
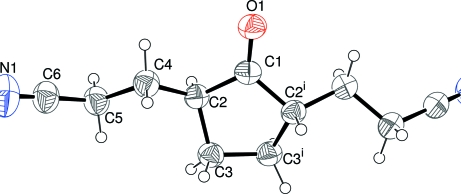
A view of the molecular structure of (I). Displacement ellopsoids are drawn at the 40% probability level and H atoms are shown as small spheres of arbitrary radius. Symmetry code: (i) 2 - x, y, 1/2 - z.

## supplementary crystallographic information

### Comment

The title compound, (I), which was first prepared by Westman & Kober (1964), is as a intermediate in the synthesis of 6,7-dihydro-5*H*-cyclopenta[*b*]pyridine ramification. We report here its structure (Fig. 1). For a related structure, see Chen *et al.* (2007).

The complete molecule of (I) is generated by crystallographic 2-fold symmetry, with the C=O group lying on the rotation axis. In the crystal, weak C—H···N interactions (Table 1) lead to zigzag chains of molecules.

### Experimental

The title compound was prepared according to the method of Westman & Kober (1964). Colourless blocks of (I) were obtained by slow evaporation of a methanol solution (m.p. 335–336 K).

### Refinement

All the H atoms were positioned geometrically (C—H = 0.97–0.98 Å), and refined as riding with *U*_iso_(H) = 1.2*U*_eq_(C).

### Crystal data

**Table:**

C_11_H_14_N_2_O	*F*_000_ = 408
*M**_r_* = 190.24	*D*_x_ = 1.161 Mg m^−^^3^
Monoclinic, *C*2/*c*	Mo *K*α radiation λ = 0.71073 Å
Hall symbol: -C 2yc	Cell parameters from 798 reflections
*a* = 18.261 (3) Å	θ = 2.9–23.6º
*b* = 7.8182 (10) Å	µ = 0.08 mm^−^^1^
*c* = 8.1943 (11) Å	*T* = 294 (2) K
β = 111.510 (9)º	Block, colorless
*V* = 1088.4 (3) Å^3^	0.24 × 0.20 × 0.10 mm
*Z* = 4	

### Data collection

**Table:**

Bruker SMART CCD diffractometer	1114 independent reflections
Radiation source: fine-focus sealed tube	644 reflections with *I* > 2σ(*I*)
Monochromator: graphite	*R*_int_ = 0.045
*T* = 294(2) K	θ_max_ = 26.4º
ω scans	θ_min_ = 2.4º
Absorption correction: multi-scan(SADABS; Bruker, 1997)	*h* = −22→14
*T*_min_ = 0.971, *T*_max_ = 0.992	*k* = −9→9
3003 measured reflections	*l* = −10→9

### Refinement

**Table:**

Refinement on *F*^2^	Secondary atom site location: difference Fourier map
Least-squares matrix: full	Hydrogen site location: inferred from neighbouring sites
*R*[*F*^2^ > 2σ(*F*^2^)] = 0.048	H-atom parameters constrained
*wR*(*F*^2^) = 0.133	*w* = 1/[σ^2^(*F*_o_^2^) + (0.0588*P*)^2^ + 0.1398*P*] where *P* = (*F*_o_^2^ + 2*F*_c_^2^)/3
*S* = 1.03	(Δ/σ)_max_ = 0.002
1114 reflections	Δρ_max_ = 0.15 e Å^−^^3^
65 parameters	Δρ_min_ = −0.13 e Å^−^^3^
Primary atom site location: structure-invariant direct methods	Extinction correction: none

### Special details

**Table:**

Geometry. All e.s.d.'s (except the e.s.d. in the dihedral angle between two l.s. planes) are estimated using the full covariance matrix. The cell e.s.d.'s are taken into account individually in the estimation of e.s.d.'s in distances, angles and torsion angles; correlations between e.s.d.'s in cell parameters are only used when they are defined by crystal symmetry. An approximate (isotropic) treatment of cell e.s.d.'s is used for estimating e.s.d.'s involving l.s. planes.
Refinement. Refinement of *F*^2^ against ALL reflections. The weighted *R*-factor *wR* and goodness of fit *S* are based on *F*^2^, conventional *R*-factors *R* are based on *F*, with *F* set to zero for negative *F*^2^. The threshold expression of *F*^2^ > σ(*F*^2^) is used only for calculating *R*-factors(gt) *etc*. and is not relevant to the choice of reflections for refinement. *R*-factors based on *F*^2^ are statistically about twice as large as those based on *F*, and *R*- factors based on ALL data will be even larger.

### Fractional atomic coordinates and isotropic or equivalent isotropic displacement parameters (Å^2^)

**Table:**

	*x*	*y*	*z*	*U*_iso_*/*U*_eq_	
O1	1.0000	1.0500 (2)	0.2500	0.0639 (6)	
N1	0.69534 (13)	0.8243 (3)	−0.3591 (3)	0.0968 (8)	
C1	1.0000	0.8948 (3)	0.2500	0.0495 (7)	
C2	0.95717 (10)	0.7841 (2)	0.0927 (2)	0.0491 (5)	
H2	0.9889	0.7818	0.0189	0.059*	
C3	0.96129 (10)	0.6064 (2)	0.1722 (2)	0.0555 (5)	
H3A	0.9174	0.5874	0.2097	0.067*	
H3B	0.9609	0.5184	0.0884	0.067*	
C4	0.87656 (11)	0.8505 (2)	−0.0189 (2)	0.0579 (6)	
H4A	0.8805	0.9706	−0.0443	0.069*	
H4B	0.8417	0.8409	0.0461	0.069*	
C5	0.84170 (11)	0.7522 (3)	−0.1902 (3)	0.0627 (6)	
H5A	0.8720	0.7769	−0.2630	0.075*	
H5B	0.8460	0.6305	−0.1651	0.075*	
C6	0.75994 (14)	0.7945 (3)	−0.2864 (3)	0.0672 (6)	

### Atomic displacement parameters (Å^2^)

**Table:**

	*U*^11^	*U*^22^	*U*^33^	*U*^12^	*U*^13^	*U*^23^
O1	0.0821 (14)	0.0465 (12)	0.0598 (12)	0.000	0.0223 (10)	0.000
N1	0.0764 (15)	0.1164 (18)	0.0812 (15)	0.0217 (12)	0.0096 (11)	−0.0174 (13)
C1	0.0539 (15)	0.0485 (17)	0.0538 (16)	0.000	0.0286 (13)	0.000
C2	0.0550 (11)	0.0493 (11)	0.0462 (11)	0.0005 (8)	0.0222 (9)	−0.0018 (8)
C3	0.0605 (11)	0.0496 (11)	0.0569 (11)	−0.0020 (9)	0.0221 (9)	−0.0027 (9)
C4	0.0598 (12)	0.0566 (12)	0.0554 (12)	0.0026 (9)	0.0191 (10)	−0.0027 (10)
C5	0.0624 (14)	0.0638 (12)	0.0570 (13)	0.0006 (10)	0.0159 (11)	−0.0072 (10)
C6	0.0680 (14)	0.0697 (15)	0.0570 (13)	0.0067 (12)	0.0147 (11)	−0.0095 (11)

### Geometric parameters (Å, °)

**Table:**

O1—C1	1.213 (3)	C3—H3A	0.9700
N1—C6	1.134 (3)	C3—H3B	0.9700
C1—C2	1.511 (2)	C4—C5	1.521 (2)
C1—C2^i^	1.511 (2)	C4—H4A	0.9700
C2—C4	1.512 (2)	C4—H4B	0.9700
C2—C3	1.525 (2)	C5—C6	1.448 (3)
C2—H2	0.9800	C5—H5A	0.9700
C3—C3^i^	1.518 (3)	C5—H5B	0.9700
			
O1—C1—C2	124.94 (10)	H3A—C3—H3B	109.0
O1—C1—C2^i^	124.95 (10)	C2—C4—C5	111.70 (15)
C2—C1—C2^i^	110.1 (2)	C2—C4—H4A	109.3
C1—C2—C4	113.82 (14)	C5—C4—H4A	109.3
C1—C2—C3	103.22 (15)	C2—C4—H4B	109.3
C4—C2—C3	117.13 (15)	C5—C4—H4B	109.3
C1—C2—H2	107.4	H4A—C4—H4B	107.9
C4—C2—H2	107.4	C6—C5—C4	112.71 (17)
C3—C2—H2	107.4	C6—C5—H5A	109.1
C3^i^—C3—C2	104.12 (10)	C4—C5—H5A	109.1
C3^i^—C3—H3A	110.9	C6—C5—H5B	109.1
C2—C3—H3A	110.9	C4—C5—H5B	109.1
C3^i^—C3—H3B	110.9	H5A—C5—H5B	107.8
C2—C3—H3B	110.9	N1—C6—C5	178.1 (3)
			
O1—C1—C2—C4	−39.96 (17)	C4—C2—C3—C3^i^	−157.50 (18)
C2^i^—C1—C2—C4	140.04 (17)	C1—C2—C4—C5	170.57 (15)
O1—C1—C2—C3	−167.96 (8)	C3—C2—C4—C5	−69.0 (2)
C2^i^—C1—C2—C3	12.04 (8)	C2—C4—C5—C6	170.72 (17)
C1—C2—C3—C3^i^	−31.6 (2)		

Symmetry codes: (i) −*x*+2, *y*, −*z*+1/2.

### Hydrogen-bond geometry (Å, °)

**Table:**

*D*—H···*A*	*D*—H	H···*A*	*D*···*A*	*D*—H···*A*
C5—H5B···N1^ii^	0.97	2.54	3.466 (3)	160

Symmetry codes: (ii) −*x*+3/2, *y*−1/2, −*z*−1/2.

## References

[bb1] Bruker (1997). *SMART*, *SAINT*, *SADABS* and *SHELXTL* Bruker AXS Inc., Madison, Wisconsin, USA.

[bb2] Chen, Y., Yang, J., Deng, Y., Li, G. & Wang, W. (2007). *Acta Cryst.* E**63**, o4054.

[bb3] Sheldrick, G. M. (2008). *Acta Cryst.* A**64**, 112–122. 10.1107/S0108767307043930 18156677

[bb4] Westman, T. L. & Kober, A. E. (1964). *J. Org. Chem* **29**, 2448–2450.

